# Supplementary material for: A Network of 17 Microtubule-Related Genes Highlights Functional Deregulations in Breast Cancer
Source: Cancers (Basel). 2023 Oct 6;15(19):4870. doi: 10.3390/cancers15194870 (PMC10571893; doi:10.3390/cancers15194870)
Supplement: Supplementary file 1 [file cancers-15-04870-s001.zip › cancers-2614605-supplementary.pdf]

Supplementary Materials

A network of 17 microtubule-related genes highlights functional deregulations in breast cancer

RODRIGUES-FERREIRA Sylvie, MORIN Morgane, GUICHAOUA Gwenn, MOINDJIE Hadia, HAYKAL Maria, COLLIER Olivier, STOVEN Veronique, NAHMIAS Clara.

Legends to Supplemental Figures

Supplemental Figure S1: **Expression of 17 MT-Rel genes in breast tumors and normal tissues.**

Proportions of tumor samples that show higher expression of the selected genes compared to adjacent normal samples at each of the quantile cutoff values (minimum Min, 1st quartile Q1, median Med, 3rd quartile Q3, maximum Max). Specificity (in red) is calculated by dividing the number of tumor samples (T) with the sum of tumor and normal samples (T+N) as described in [1].

Supplemental Figure S2: Expression of 17 MT-Rel genes in breast tumors according to molecular subtypes.

Probeset intensities of each appropriate gene in breast tumors from the REMAGUS02 cohort, classified according to molecular subtypes: ER+ (ER-positive), HER2+ (HER2-overexressing) and TNBC (Triple negative breast cancer subtype). A blue line indicates the median value. *p<0.05; **p<0.01; ***p<0.001; ****p<0.0001.

Supplemental Figure S3: Survival curves of breast cancer patients according to MT-Rel gene expression level.

(A) Overall survival curves of breast cancer patients according to the 17 MT-Rel genes probeset intensities from kmplotter (kmplot.com). **(B)** Relapse-free survival curves as in **(A).** For each gene, the best probeset was used (Jetset) and the best cut-off was selected to distinguish between tumors expressing low and high levels of the gene.

Legends to Supplemental Tables

Supplemental Table S1. Differential expression of 17 MT-Rel genes in breast tumor samples relative to adjacent normal tissues.

Median fold change and associated Mann-Whitney p-value were determined for each MT-Rel gene in 112 breast tumors and adjacent normal tissues from TNMplot.org.

Supplemental Table S2. Pearson's correlation (r) values and associated p-values in normal breast tissues.

p-values that do not reach significance are indicated in grey. Genes are ordered according to their fold change in breast tumor vs normal tissue as shown in Fig. 1B.

Supplemental Table S3. Pearson's correlation (r) values and associated p-values in breast tumors.

p values that do not reach significance are indicated in grey. Genes are ordered as in Suppl Table S2.

Supplemental Table S4. Overall survival (OS) and Relapse-free survival (RFS) analysis in breast cancer patients.

Best probeset and best cut-off for each gene were determined from kmplot.com. HR: Hazard Ratio; CI: Confidence Interval; n: number of patients analyzed. Genes are ordered as in Suppl Table S2.

Supplemental Table S5. Functional consequences of MT-Rel silencing on breast cancer cell viability.

Mean fold change and standard error mean (sem) in cell viability of breast cancer cell lines MDA-MB-231 and MDA-MB-468 following silencing of 14 overexpressed MT-Rel genes by siRNA screening. Significant p-values are indicated in blue. Shown are the results of four independent experiments performed in quadruplicate. Results are normalized to control siRNA.

Supplemental Table S6. DepMap analysis of MT-Rel gene depletion on viability of 47 breast cancer cell lines.

Chronos dependency score values across the 47 breast cancer cell lines. Genes are presented by alphabetical order.

Reference

1. Bartha, Á.; Győrffy, B. TNMplot.com: A Web Tool for the Comparison of Gene Expression in Normal, Tumor and Metastatic Tissues. *Int. J. Mol. Sci.* **2021**, *22*, doi:10.3390/ijms22052622.

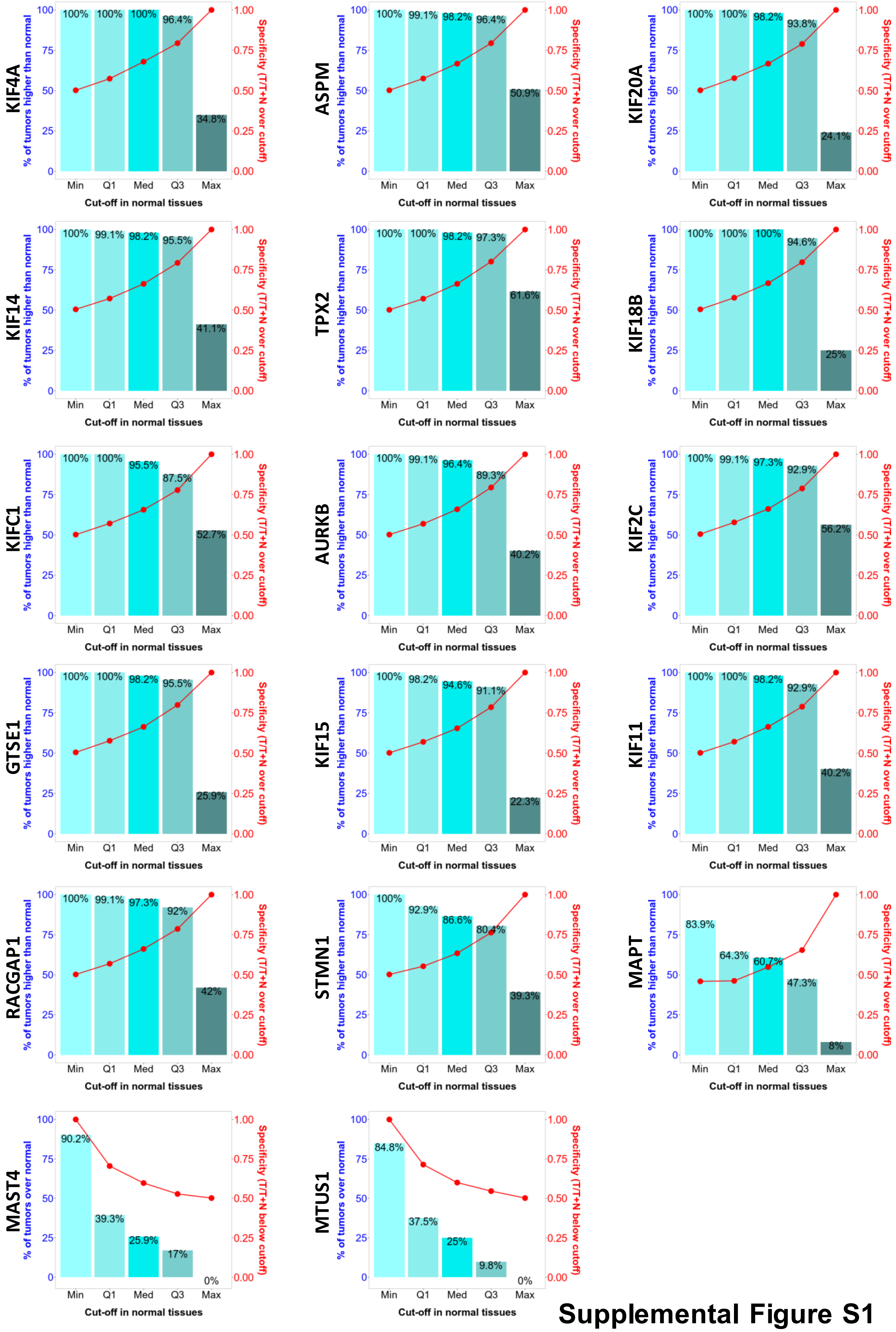

Supplemental Figure S1

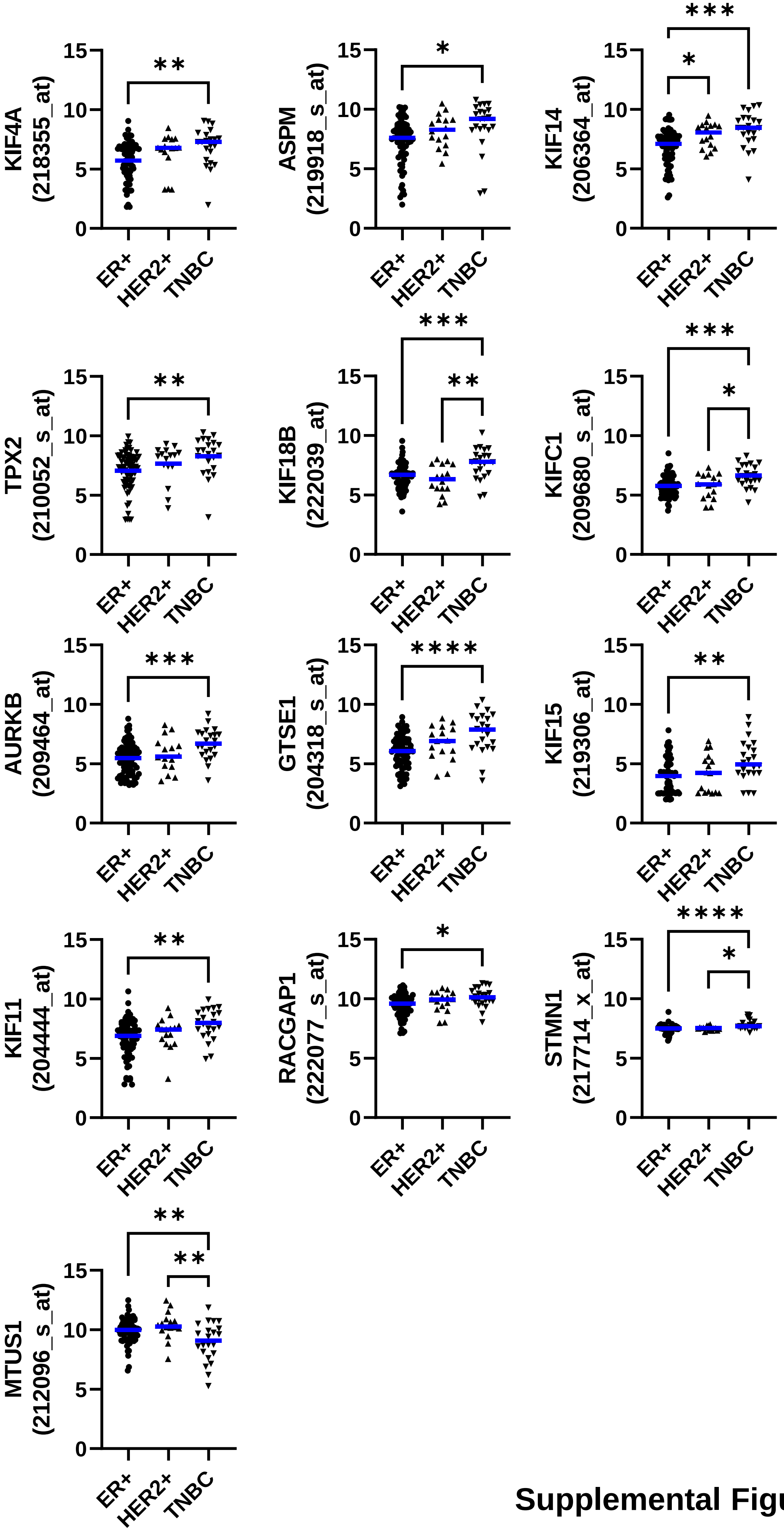

Supplemental Figure S2

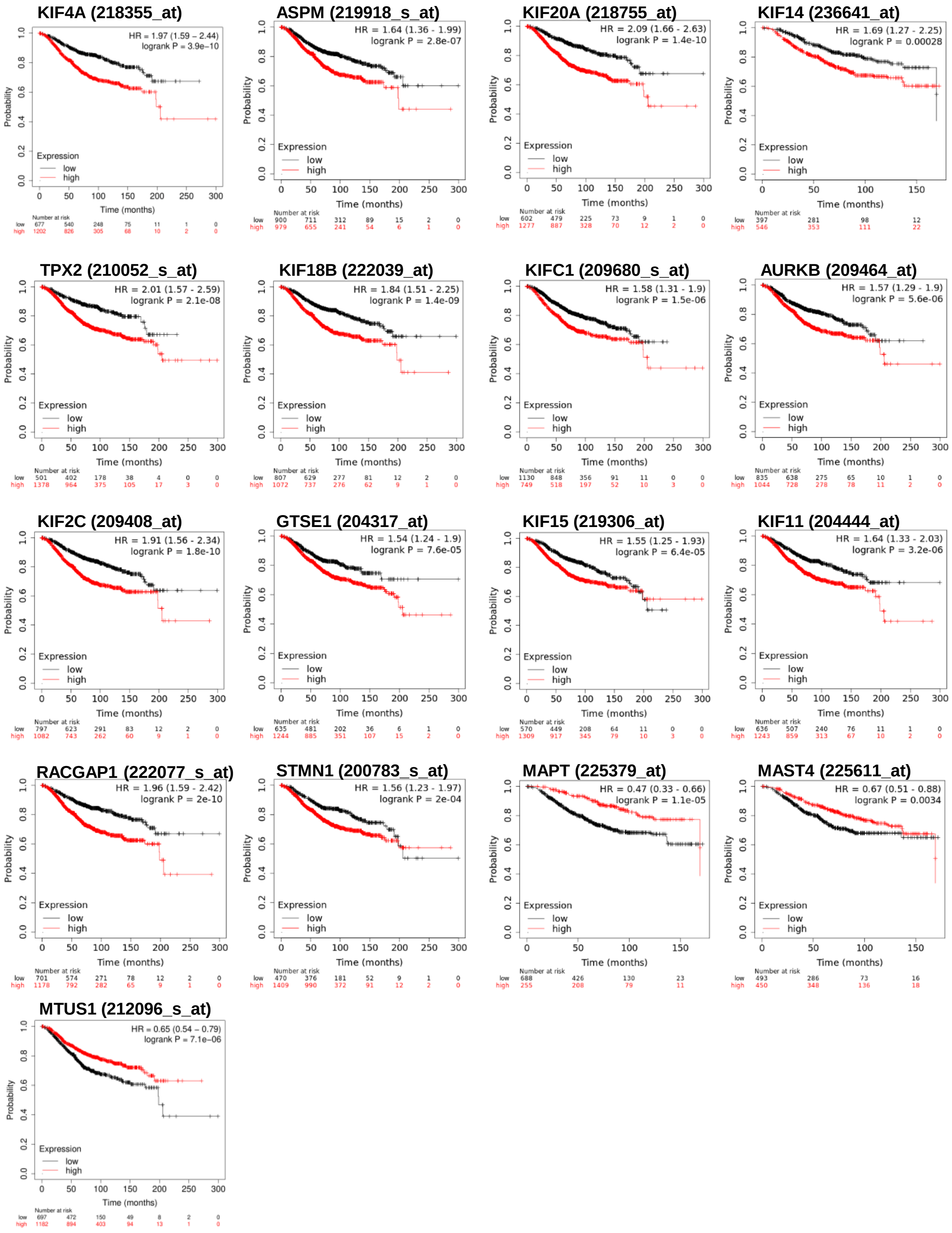

Supplemental Figure S3A

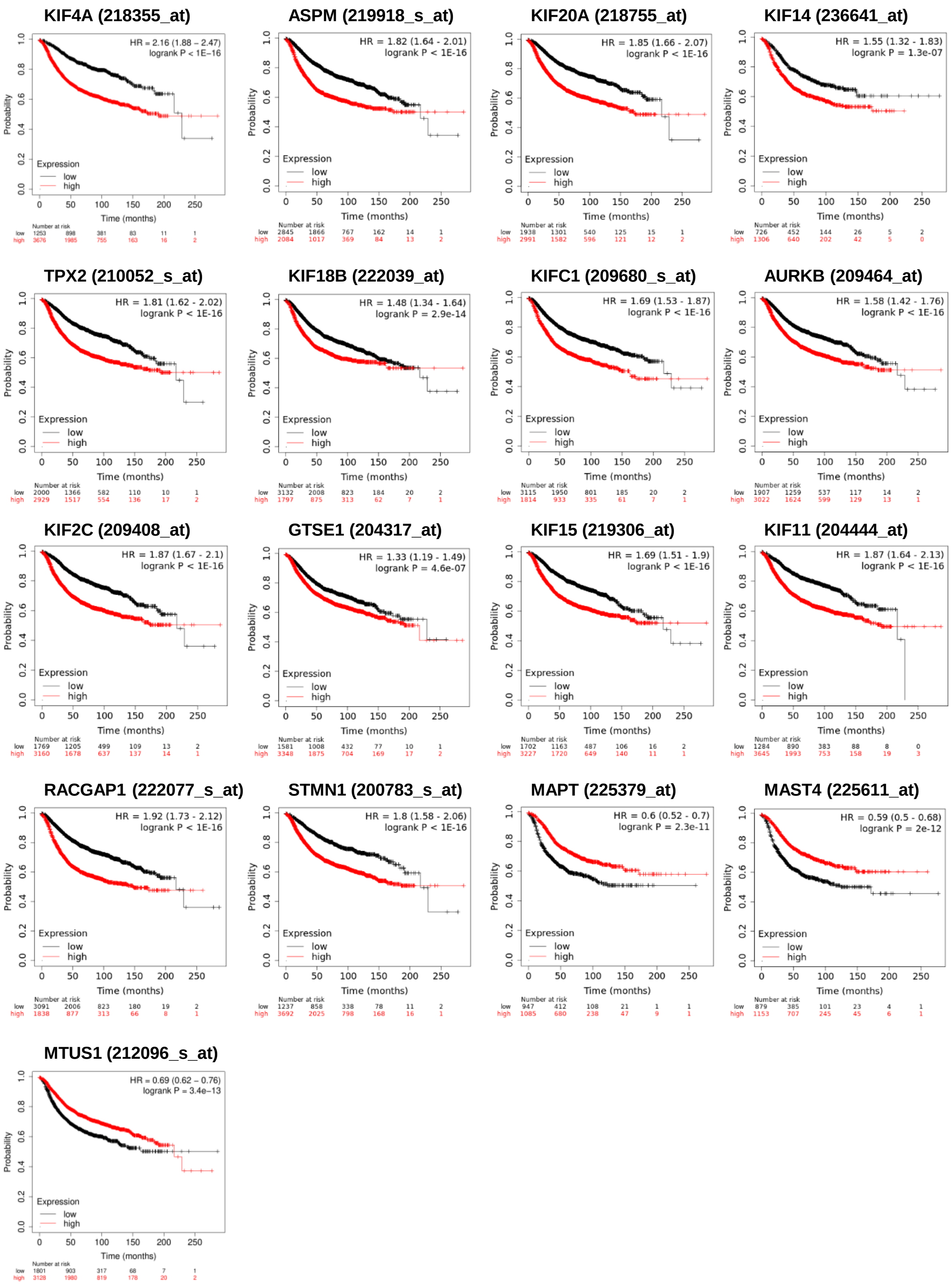

Supplemental Figure S3B

Gene	Fold change (Median)	Mann-Whitney p-value
**KIF4A**	17,79	$8,57.10^{-20}$
**ASPM**	14,66	$9,67.10^{-20}$
**KIF20A**	13,01	$3,59.10^{-19}$
**KIF14**	12,27	$4,05.10^{-19}$
**TPX2**	11,72	$4,38.10^{-20}$
**KIF18B**	11,45	$5,36.10^{-20}$
**KIFC1**	8,19	$6,22.10^{-20}$
**AURKB**	8,05	$5,98.10^{-19}$
**KIF2C**	7,89	$4,88.10^{-20}$
**GTSE1**	7,16	$1,81.10^{-19}$
**KIF15**	5,45	$7,97.10^{-19}$
**KIF11**	5,18	$3,23.10^{-19}$
**RACGAP1**	3,61	$2,12.10^{-19}$
**STMN1**	2,52	$3,39.10^{-17}$
**MAPT**	1,44	$8,46.10^{-4}$
**MAST4**	0,68	$1,39.10^{-4}$
**MTUS1**	0,66	$1,39.10^{-6}$

Supplemental Table S1: Differential expression of 17 MT-Rel genes in breast tumor samples relative to adjacent normal tissues.

(A) Pearson (r)	KIF4A	ASPM	KIF20A	KIF14	TPX2	KIF18B	KIFC1	AURKB	KIF2C	GTSE1	KIF15	KIF11	RACGAP1	STMN1	MAPT	MAST4	MTUS1
KIF4A	1	0,819	0,890	0,854	0,795	0,938	0,745	0,715	0,762	0,817	0,880	0,696	0,707	0,377	0,175	- 0,177	- 0,115
ASPM		1	0,795	0,842	0,865	0,809	0,718	0,780	0,748	0,764	0,834	0,743	0,679	0,381	0,114	- 0,230	- 0,066
KIF20A			1	0,780	0,796	0,922	0,764	0,753	0,731	0,848	0,878	0,699	0,694	0,366	0,143	- 0,203	- 0,104
KIF14				1	0,748	0,800	0,653	0,610	0,630	0,696	0,814	0,655	0,720	0,446	0,147	- 0,131	- 0,086
TPX2					1	0,814	0,726	0,743	0,808	0,790	0,798	0,712	0,660	0,338	0,159	- 0,263	- 0,148
KIF18B						1	0,776	0,797	0,752	0,883	0,911	0,720	0,677	0,359	0,150	- 0,211	- 0,115
KIFC1							1	0,736	0,739	0,758	0,751	0,702	0,595	0,477	0,240	- 0,199	- 0,070
AURKB								1	0,748	0,819	0,799	0,780	0,492	0,479	0,073	- 0,309	- 0,110
KIF2C									1	0,752	0,713	0,672	0,614	0,362	0,205	- 0,234	- 0,118
GTSE1										1	0,845	0,709	0,572	0,366	0,096	- 0,191	- 0,149
KIF15											1	0,793	0,646	0,377	0,118	- 0,143	- 0,060
KIF11												1	0,548	0,318	0,192	- 0,211	0,015
RACGAP1													1	0,246	0,233	- 0,101	- 0,027
STMN1														1	- 0,012	- 0,109	- 0,100
MAPT															1	- 0,077	0,119
MAST4																1	0,027
MTUS1																	1

(B) p values	KIF4A	ASPM	KIF20A	KIF14	TPX2	KIF18B	KIFC1	AURKB	KIF2C	GTSE1	KIF15	KIF11	RACGAP1	STMN1	MAPT	MAST4	MTUS1
KIF4A		2,76E-28	2,18E-39	5,54E-33	1,13E-25	2,13E-52	4,66E-21	7,86E-19	1,81E-22	4,16E-28	2,27E-37	1,59E-17	2,85E-18	4,19E-05	0,06	0,06	0,23
ASPM			1,18E-25	2,76E-31	1,00E-34	4,10E-27	4,87E-19	4,27E-24	2,44E-21	1,09E-22	3,31E-30	7,15E-21	1,99E-16	3,32E-05	0,23	0,01	0,49
KIF20A				4,29E-24	1,06E-25	4,64E-47	1,20E-22	9,95E-22	5,43E-20	3,59E-32	4,71E-37	9,94E-18	2,21E-17	7,31E-05	0,13	0,03	0,28
KIF14					2,43E-21	3,63E-26	6,06E-15	9,24E-13	9,88E-14	1,52E-17	9,10E-28	4,80E-15	3,71E-19	8,45E-07	0,12	0,17	0,37
TPX2						1,17E-27	1,28E-19	6,36E-21	4,65E-27	4,17E-25	5,87E-26	1,38E-18	2,63E-15	2,69E-04	0,09	0,01	0,12
KIF18B							8,52E-24	7,57E-26	1,26E-21	5,09E-38	3,45E-44	3,92E-19	2,46E-16	1,04E-04	0,11	0,03	0,23
KIFC1								2,32E-20	1,42E-20	4,22E-22	1,60E-21	6,43E-18	4,66E-12	1,08E-07	0,01	0,04	0,46
AURKB									2,83E-21	2,84E-28	4,47E-26	4,15E-24	3,54E-08	9,26E-08	0,44	9,09E-04	0,25
KIF2C										1,22E-21	1,15E-18	5,08E-16	5,83E-13	8,69E-05	0,03	0,01	0,22
GTSE1											1,01E-31	2,19E-18	4,59E-11	7,07E-05	0,31	0,04	0,12
KIF15												1,86E-25	1,42E-14	4,25E-05	0,22	0,13	0,53
KIF11													3,94E-10	6,31E-04	0,04	0,03	0,88
RACGAP1														8,94E-03	0,01	0,29	0,78
STMN1															0,90	0,25	0,29
MAPT																0,42	0,21
MAST4																	0,78
MTUS1																	

Supplemental Table S2: Pearson's correlation (r) values (**A**) and associated p-values (**B**) in normal breast tissues.

(A) Pearson (r)	KIF4A	ASPM	KIF20A	KIF14	TPX2	KIF18B	KIFC1	AURKB	KIF2C	GTSE1	KIF15	KIF11	RACGAP1	STMN1	MAPT	MAST4	MTUS1
KIF4A	1	0,558	0,602	0,412	0,627	0,641	0,600	0,628	0,722	0,538	0,649	0,647	0,555	0,575	-0,143	-0,101	-0,225
ASPM		1	0,558	0,531	0,428	0,504	0,500	0,441	0,607	0,548	0,533	0,568	0,347	0,532	-0,177	-0,038	-0,187
KIF20A			1	0,449	0,553	0,612	0,545	0,596	0,665	0,463	0,610	0,515	0,470	0,667	-0,162	-0,113	-0,284
KIF14				1	0,459	0,416	0,437	0,460	0,459	0,593	0,487	0,576	0,472	0,389	-0,076	-0,057	-0,116
TPX2					1	0,683	0,632	0,794	0,716	0,447	0,644	0,639	0,520	0,590	-0,190	-0,159	-0,222
KIF18B						1	0,714	0,671	0,703	0,468	0,781	0,566	0,536	0,538	-0,057	0,009	-0,236
KIFC1							1	0,626	0,598	0,581	0,673	0,557	0,524	0,482	-0,145	-0,068	-0,240
AURKB								1	0,725	0,464	0,640	0,619	0,547	0,644	-0,268	-0,280	-0,298
KIF2C									1	0,563	0,697	0,588	0,494	0,785	-0,119	-0,230	-0,289
GTSE1										1	0,501	0,497	0,389	0,441	-0,123	-0,143	-0,134
KIF15											1	0,675	0,632	0,591	-0,029	0,020	-0,165
KIF11												1	0,665	0,491	-0,132	0,074	-0,203
RACGAP1													1	0,468	-0,044	-0,070	-0,180
STMN1														1	-0,154	-0,157	-0,286
MAPT															1	0,372	0,082
MAST4																1	0,029
MTUS1																	1

(B) p values	KIF4A	ASPM	KIF20A	KIF14	TPX2	KIF18B	KIFC1	AURKB	KIF2C	GTSE1	KIF15	KIF11	RACGAP1	STMN1	MAPT	MAST4	MTUS1
KIF4A		1,65E-10	2,13E-12	6,42E-06	1,33E-13	2,58E-14	2,70E-12	1,22E-13	2,69E-19	9,30E-10	1,02E-14	1,26E-14	2,11E-10	3,51E-11	0,133	0,289	0,017
ASPM			1,68E-10	1,78E-09	2,50E-06	1,50E-08	1,96E-08	1,12E-06	1,31E-12	3,89E-10	1,50E-09	6,62E-11	1,82E-04	1,58E-09	0,061	0,691	0,048
KIF20A				7,04E-07	2,57E-10	7,82E-13	5,38E-10	4,24E-12	1,21E-15	2,81E-07	9,74E-13	6,17E-09	1,74E-07	9,98E-16	0,088	0,236	0,002
KIF14					3,63E-07	5,01E-06	1,50E-06	3,35E-07	3,67E-07	5,60E-12	5,11E-08	3,11E-11	1,49E-07	2,24E-05	0,428	0,552	0,223
TPX2						1,11E-16	7,50E-14	1,51E-25	7,68E-19	7,88E-07	1,96E-14	3,42E-14	4,16E-09	7,87E-12	0,045	0,093	0,019
KIF18B							9,14E-19	5,96E-16	5,74E-18	1,93E-07	3,04E-24	7,89E-11	1,11E-09	9,22E-10	0,549	0,925	0,012
KIFC1								1,65E-13	3,39E-12	1,92E-11	4,12E-16	1,84E-10	3,10E-09	7,50E-08	0,127	0,475	0,011
AURKB									1,48E-19	2,50E-07	3,12E-14	3,38E-13	4,19E-10	1,94E-14	0,004	0,003	0,001
KIF2C										1,07E-10	1,42E-17	9,23E-12	3,15E-08	1,16E-24	0,211	0,015	0,002
GTSE1											1,89E-08	2,41E-08	2,19E-05	1,11E-06	0,197	0,133	0,158
KIF15												3,20E-16	7,47E-14	6,96E-12	0,759	0,835	0,081
KIF11													1,21E-15	3,97E-08	0,166	0,436	0,032
RACGAP1														1,92E-07	0,648	0,466	0,057
STMN1															0,104	0,097	0,002
MAPT																0,000	0,393
MAST4																	0,759
MTUS1																	

Supplemental Table S3: Pearson's correlation (r) values (**A**) and associated p-values (**B**) in breast tumors.

		OS				RFS				Bad Prognosis
**Gene**	**Affy ID**	**HR**	**CI**	**P value**	**n**	**HR**	**CI**	**P value**	**n**	**Expression level**
**KIF4A**	218355_at	1,97	1,59-2,44	3.9e-10	1879	2,16	1,88-2,47	<1e-16	4929	High
**ASPM**	219918_s_at	1,64	1,36-1,99	2.8e-7	1879	1,82	1,64-2,01	<1e-16	4929	High
**KIF20A**	218755_at	2,09	1,66-2,63	1.4e-10	1879	1,85	1,66-2,07	<1e-16	4929	High
**KIF14**	236641_at	1,69	1,27-2,25	2.8e-4	943	1,55	1,32-1,83	1.3e-7	2032	High
**TPX2**	210052_s_at	2,01	1,57-2,59	2.1e-8	1879	1,81	1,62-2,02	<1e-16	4929	High
**KIF18B**	222039_at	1,84	1,51-2,25	1.4e-9	1879	1,48	1,34-1,64	2.9e-14	4929	High
**KIFC1**	209680_s_at	1,58	1,31-1,9	1.5e-6	1879	1,69	1,53-1,87	<1e-16	4929	High
**AURKB**	209464_at	1,57	1,29-1,9	5.6e-6	1879	1,58	1,42-1,76	<1e-16	4929	High
**KIF2C**	209408_at	1,91	1,56-2,34	1.8e-10	1879	1,87	1,67-2,1	<1e-16	4929	High
**GTSE1**	204317_at	1,54	1,24-1,9	7.6e-5	1879	1,33	1,19-1,49	4.6e-7	4929	High
**KIF15**	219306_at	1,55	1,25-1,93	6.4e-5	1879	1,69	1,51-1,9	<1e-16	4929	High
**KIF11**	204444_at	1,64	1,33-2,03	3.2e-6	1879	1,87	1,64-2,13	<1e-16	4929	High
**RACGAP1**	222077_s_at	1,96	1,59-2,42	2.0e-10	1879	1,92	1,73-2,12	<1e-16	4929	High
**STMN1**	200783_s_at	1,56	1,23-1,97	2.0e-4	1879	1,8	1,58-2,06	<1e-16	4929	High
**MAPT**	225379_at	0,47	0,33-0,66	1.1e-5	943	0,6	0,52-0,7	2.3e-11	2032	Low
**MAST4**	225611_at	0,67	0,51-0,88	0.0034	943	0,59	0,5-0,68	2.0e-12	2032	Low
**MTUS1**	212096_s_at	0,65	0,54-0,79	7.1e-6	1879	0,69	0,62-0,76	3.4e-13	4929	Low

Supplemental Table S4: Overall survival (OS) and Relapse-free survival (RFS) analysis in breast cancer patients.

	MDA-MB-231			MDA-MB-468		
	mean	sem	pvalue	mean	sem	pvalue
**KIF4A**	1,042	0,049	0,9995	1,017	0,094	0,9999
**ASPM**	0,768	0,042	0,4024	**0,472**	**0,020**	**0,0338**
**KIF20A**	0,977	0,103	0,9997	0,889	0,220	0,999
**KIF14**	0,930	0,093	0,9991	0,856	0,158	0,9892
**TPX2**	**0,395**	**0,026**	**0,0001**	**0,247**	**0,022**	**0,0008**
**KIF18B**	0,803	0,088	0,5984	1,080	0,252	0,9993
**KIFC1**	**0,410**	**0,086**	**0,0002**	**0,253**	**0,097**	**0,0009**
**AURKB**	**0,430**	**0,097**	**0,0003**	**0,298**	**0,069**	**0,002**
**KIF2C**	0,807	0,055	0,6272	0,545	0,073	0,0942
**GTSE1**	0,849	0,114	0,8637	0,536	0,074	0,0832
**KIF15**	0,868	0,132	0,9366	**0,373**	**0,094**	**0,0071**
**KIF11**	**0,312**	**0,100**	**<0,0001**	**0,018**	**0,006**	**<0,0001**
**RACGAP1**	**0,621**	**0,111**	**0,0317**	0,852	0,183	0,9876
**STMN1**	0,715	0,081	0,1841	0,561	0,072	0,1151

Supplemental Table S5: Functional consequences of MT-Rel silencing on breast cancer cell viability.

Breast cancer cell line	DepMap_ID	ASPM	AURKB	GTSE1	KIF2C	KIF4A	KIF11	KIF14	KIF15	KIF18B	KIF20A	KIFC1	RACGAP1	STMN1	TPX2
**21NT**	ACH-002399	-0,43	-2,10	-0,21	-0,49	-0,77	-2,97	-0,34	-0,24	-0,48	-0,49	-0,48	-1,81	0,09	-1,91
**AU565**	ACH-000248	-0,12	-2,65	-0,02	-0,35	-0,73	-2,48	-0,44	-0,10	-0,31	-0,35	-0,06	-1,33	0,15	-2,06
**BT549**	ACH-000288	-0,06	-2,90	-0,13	-0,50	-0,55	-2,70	-0,39	-0,26	-0,95	-0,75	-0,90	-1,72	0,03	-1,07
**CAL120**	ACH-000212	-0,85	-2,63	-0,04	-0,47	-0,47	-3,17	-0,14	-0,08	-0,42	-0,74	-0,25	-1,51	0,08	-1,75
**CAL51**	ACH-000856	-0,32	-2,15	-0,24	-0,32	-0,50	-2,50	-0,58	-0,21	-0,84	-0,68	-0,13	-1,40	-0,03	-1,16
**CAMA1**	ACH-000783	0,02	-2,52	-0,25	-0,14	-0,60	-3,47	-0,77	-0,15	-0,59	-0,79	-0,10	-1,27	0,06	-1,89
**DU4475**	ACH-000258	0,00	-1,49	-0,06	-0,90	-0,34	-2,59	-0,27	-0,10	-1,01	-0,62	-0,34	-1,72	-0,01	-1,86
**EFM19**	ACH-000330	-0,08	-1,62	-0,68	-0,18	-0,33	-3,17	-0,36	-0,26	-0,13	-0,45	-0,33	-1,34	0,08	-1,17
**EVSAT**	ACH-001065	-0,33	-2,58	-0,48	-1,28	-0,51	-2,84	-0,29	-0,09	-1,11	-0,72	0,02	-2,40	0,01	-0,75
**HCC1143**	ACH-000374	-0,30	-1,93	-0,35	-0,51	-0,09	-3,27	-0,57	-0,42	-0,77	-0,49	-0,29	-1,31	-0,13	-1,23
**HCC1187**	ACH-000111	-0,09	-2,91	-0,26	-0,40	-0,36	-2,32	-0,43	0,40	-0,05	-0,78	0,13	-1,82	0,25	-1,15
**HCC1395**	ACH-000699	-0,52	-1,98	-0,40	-0,48	-0,80	-3,81	-0,14	-0,40	-0,43	-0,94	-0,21	-1,61	-0,10	-1,93
**HCC1419**	ACH-000277	-0,16	-2,03	-0,10	-0,34	-0,83	-2,54	-1,17	-0,14	-0,25	-0,60	-0,30	-1,29	0,29	-1,00
**HCC1428**	ACH-000352	-0,50	-2,22	-0,12	-0,16	-0,50	-3,07	-0,30	0,01	-0,21	-0,40	-0,09	-1,42	-0,24	-1,46
**HCC1806**	ACH-000624	-0,29	-2,99	-0,11	-0,15	-0,36	-3,35	-0,62	-0,20	-0,25	-0,81	-0,06	-1,61	0,00	-1,15
**HCC1937**	ACH-000223	-0,30	-2,09	-0,18	-0,53	-0,53	-2,75	-0,61	-0,20	-0,49	-0,94	-0,17	-1,33	-0,01	-0,99
**HCC1954**	ACH-000859	-0,23	-2,05	-0,33	-0,17	-0,76	-3,25	-0,51	-0,21	-0,24	-0,51	-0,54	-1,52	0,13	-1,54
**HCC202**	ACH-000725	-0,34	-2,18	-0,39	-0,07	-1,04	-3,59	-0,25	-0,29	-0,40	-0,52	-0,17	-1,48	-0,04	-2,38
**HCC38**	ACH-000276	-0,26	-3,04	-0,79	-0,32	-0,74	-3,28	-1,11	-0,55	-0,47	-0,80	-0,08	-1,78	0,13	-1,90
**HCC70**	ACH-000668	-0,79	-3,10	-0,21	-0,55	-0,46	-3,08	-0,68	-0,12	-0,56	-1,00	-0,24	-2,01	-0,18	-1,47
**HMC18**	ACH-000721	-0,46	-2,59	-0,09	-0,35	-0,56	-2,65	-0,65	-0,06	-0,33	-0,56	-0,29	-1,43	0,10	-1,00
**HS578T**	ACH-000148	-0,26	-2,35	-0,24	-0,51	-0,50	-3,15	-0,29	-0,21	-0,45	-0,36	-0,18	-1,48	0,05	-1,09
**JIMT1**	ACH-000711	-0,45	-2,54	-0,32	-0,25	-0,24	-2,45	-0,40	-0,24	-0,20	-0,46	-0,41	-1,52	0,05	-0,87
**KPL1**	ACH-000028	-0,63	-2,15	-0,04	-0,32	-0,80	-1,83	-0,48	-0,08	-0,45	-0,35	-0,22	-1,52	0,00	-1,70
**MCF7**	ACH-000019	-0,41	-1,91	0,01	-0,21	-0,55	-2,84	-0,77	-0,16	-0,73	-0,65	-0,06	-1,49	-0,05	-1,69
**MDAMB157**	ACH-000621	-0,54	-2,74	-0,20	-0,37	-1,51	-3,03	-0,46	-0,02	-0,18	-0,76	-0,88	-1,66	0,01	-1,72
**MDAMB231**	ACH-000768	-0,36	-2,15	-0,01	-0,18	-0,31	-2,27	-0,30	-0,06	-0,32	-0,82	-0,25	-1,38	0,06	-0,87
**MDAMB361**	ACH-000934	-0,45	-2,31	-0,11	-0,37	-0,54	-2,98	-0,27	0,05	-0,37	-0,55	-0,03	-0,90	0,09	-3,29
**MDAMB415**	ACH-000876	0,00	-2,32	-0,19	-0,01	-0,51	-3,70	-0,35	0,01	-0,40	-0,20	-0,11	-1,78	0,11	-2,25
**MDAMB436**	ACH-000573	-0,13	-1,80	-0,35	-0,46	-0,43	-3,11	-0,42	-0,16	-0,62	-0,17	-0,23	-1,71	0,08	-1,13
**MDAMB453**	ACH-000910	-0,05	-2,31	-0,34	-0,38	-1,07	-3,70	-0,46	-0,08	-0,63	-0,41	-0,19	-1,36	-0,01	-2,14
**MDAMB468**	ACH-000849	-0,13	-2,92	-0,13	-0,27	0,03	-3,46	-0,90	-0,16	-0,12	-0,78	-0,20	-1,68	-0,35	-1,25
**MFM223**	ACH-001819	-0,94	-2,60	-0,50	-0,24	-0,43	-3,30	-0,89	-0,35	-0,22	-0,52	-0,02	-1,67	-0,02	-1,47
**OCUBM**	ACH-002179	-1,33	-2,56	0,14	-0,60	-0,98	-2,75	-1,18	0,09	-0,19	-0,88	0,09	-2,31	0,05	-2,02
**SKBR3**	ACH-000017	-0,58	-2,21	-0,17	-0,02	-0,46	-3,00	-0,47	-0,10	-0,22	-0,87	-0,19	-1,47	0,05	-1,03
**SUM102PT**	ACH-001388	-0,04	-2,16	-0,07	-0,48	-0,89	-3,25	-0,44	-0,15	-0,57	-0,53	-0,14	-1,28	-0,04	-1,43
**SUM1315MO2**	ACH-001389	-0,08	-3,20	-0,01	-0,29	-0,38	-2,84	-0,22	-0,38	-0,17	-0,67	-0,09	-1,35	-0,10	-1,28
**SUM149PT**	ACH-001390	-0,19	-1,82	0,06	-0,53	-0,47	-2,68	-0,41	0,03	-0,51	-0,64	-0,59	-0,91	-0,10	-0,77
**SUM159PT**	ACH-001391	-0,42	-2,38	-0,06	-0,09	-0,42	-3,02	-0,55	-0,15	-0,38	-1,29	-0,43	-1,63	-0,16	-1,60
**SUM185PE**	ACH-001392	-0,33	-2,30	-0,13	0,02	-0,38	-2,56	-0,66	-0,14	-0,26	-0,97	-0,11	-1,26	0,04	-1,57
**SUM190PT**	ACH-001393	-0,12	-1,74	-0,15	-0,27	-0,63	-3,64	-0,39	-0,12	-0,19	-0,46	-0,04	-1,23	0,11	-1,43
**SUM229PE**	ACH-001394	-0,16	-3,07	-0,60	-0,34	-0,47	-2,24	-0,47	-0,25	-0,47	-0,71	-0,03	-1,94	-0,18	-0,81
**SUM52PE**	ACH-001396	-0,34	-1,51	-0,18	-0,53	-0,53	-3,14	-0,52	-0,12	-0,75	-0,55	-0,23	-0,73	-0,07	-2,20
**T47D**	ACH-000147	0,11	-2,05	-0,14	-0,08	-0,29	-2,79	-0,38	-0,16	-0,26	-0,32	-0,06	-1,35	0,11	-1,44
**UACC893**	ACH-000554	-0,40	-0,84	-0,12	-0,22	-1,28	-3,87	-0,53	-0,07	-0,21	-0,46	-0,02	-0,55	0,22	-2,59
**VP229**	ACH-001419	-0,25	-1,93	-0,30	-0,44	-0,41	-2,94	-0,13	-0,29	-0,32	-0,72	-0,32	-1,42	0,05	-1,40
**ZR751**	ACH-000097	-0,22	-2,37	-0,21	-0,64	-0,71	-3,18	-0,43	-0,18	-0,68	-0,54	-0,08	-1,37	-0,25	-1,96

Supplemental Table S6: DepMap analysis of MT-Rel gene depletion on viability of 47 breast cancer cell lines.
